# Tissue surface adaptation and retention of digital obturator after one year of use

**DOI:** 10.1186/s12903-024-04639-2

**Published:** 2024-08-07

**Authors:** Khadija Mohamed Abd El Salam khalaf, Hoda Mohamed Amin Rashad, Tamer Mohamed Nasr Mostafa

**Affiliations:** Prosthodontic Department, Faculty of Dentistry, Tanta University, Elgeish st. Tanta, Tanta, Egypt

**Keywords:** CAD_CAM, Digital obturator, Tissue adaptation

## Abstract

**Background:**

Effect of aging on tissue adaptability and retention of digital obturator is still under investigation.

**Methods:**

A maxillary Armany (class I) epoxy reference model was scanned to fabricate digital obturator fabricated from milled Co-Cr framework and 3D printed bulb. A color map of the scanned reference and digital obturator was made using Geomagic software to evaluate the accuracy of fit before and after cyclic loading using ROBOTA chewing simulator at 37,500, 75,000 and 150,000 cycles to simulate clinically 3-, 6- and 12-months chewing condition. Insertion-removal condition simulating the placement and removal of the obturator was done using repeated 360, 720 and 1440 cycles and retention was evaluated before and after the repeated cycles. Data were collected, tabulated and statistically analyzed using Statistical Package for Social Sciences (IBM SPSS Statistics 26). Student t-test and multi variable ANOVA test were used to detect significance. P-value < 0.05 was considered significant difference.

**Results:**

For retention test: There was a significant difference between baseline and 3, 6 and12 months. For the tissue surface adaptation test: There was significant difference at all measured areas (P-value < 0.05) before and after application of load.

**Conclusion:**

digitally designed and fabricated obturator was highly retentive and has excellent tissue surface adaptation upon fabrication, After application of load; reduction of retention and lack of tissue adaptation were resulted.

**The clinical implication:**

of this manuscript is that digital obturator can be used successfully with the shortcomings of loosening retention and adaptation afterwhile. So, clinical trials should investigate the clinical acceptance of these shortcomings.

## Background

Maxillofacial defects may result from congenital or acquired defects such as trauma, tumor, pathologic changes, radiation and burns. Acquired palatal defects may affect anatomical structures resulting in oro-antral communication and sometimes the nasal cavity as well [1].

Several trials to change the conventional procedures of maxillofacial obturator construction from being time-consuming, skillful operator-dependent and uncomfortable for patients to be digitalized and more simplified procedures using CAD/ CAM technology were reported earlier. [2–4]

To overcome the disadvantages of traditional method, the full digitalization is used which leads to favorable clinical outcomes, better retention, fewer patient visits, better material properties and biocompatibility. identification of anatomical landmarks on digitized casts, easy data storage and production of duplicated obturators, reduce hazards of infectious cross contamination, It allows application of provisions for esthetic designs, excellent precision of fit and longevity [4].

Conventional impression has been the standard for years but dimensional changes in dental stones and the volumetric changes of impression materials lead to inaccuracies. So Digital scanners and CAD-CAM (computer-aided design and manufacturing) technologies were created to overcome these challenges with conventional approaches [5].

With the progress in digital technology, there were several trials for reconstruction of maxillofacial defects to improve appearance and function with more accurate surgery and shorter operation times. With CAD/CAM software, accurate pre-operative planning can be designed, surgeons can perform virtual osteotomy and reconstruction procedures, overcoming the disadvantages of autogenous bone grafts, and performing resection and reconstruction in one step or create patient-specific implant with accurate fitting of implants.(84–85) The 3D printing technique in the maxillofacial area includes models that are more accurate replicas of patient-specific anatomy with more accurate esthetic results and restoration of complex anatomical defects [2].

A CAD-CAM-derived prosthesis has to undergo multiple steps like data collection, data processing and manufacturing that affect the fit of the prosthesis .There are two possibilities to analyze the accuracy of data acquired from intraoral and extraoral scanners. Firstly, to compare the fit of the restoration or to compare the surface tessellation language (STL) datasets with a reference dataset [5].

Manufacturing is the last phase of dental CAD/CAM process. It helps to get a restoration from a CAD model that undergoes processing, finishing, and polishing before being inserted into the patient mouth. There are two main fabrication methods which are subtractive SM (milling or grinding), or additive manufacturing (AM) is often referred as “layered manufacturing,” “direct digital manufacturing,” “three-dimensional printing,” “solid freeform fabrication” or “generative manufacturing techniques” and known as rapid prototyping or 3D printing[4].

Fabrication of an obturator for the maxillary defect provides major challenge because of the lack of support from teeth and lack of stability and retention. In addition, excess resorption of the residual edentulous ridges affect adaptation of obturator [6].

By aging, there is decrease in retention that may be due to withdrawal of an obturator from the defect area greatly reduces the retention of buccally placed clasps specially in cases with fewer teeth, less tissue to support the prosthesis, and a larger area of the defect [7]. A previous study showed that obturators framework designs with palatal plating on all teeth and the major connector for all designs was made to extend into the defect facilitated load transmission to the model so get better retention and adaptation [7].

The authors determined that there was a change in the retentive force of the Co- Cr alloy clasps after repeated cycling loads of simulated placement and removal. In contrast, a study showed that after a test simulating 5 years of service, Co-Cr alloy clasps showed a residual retentive force to satisfy the requirements for clinical use [8].

A previous study reported that misfit most commonly occurs in maxillary framework specially in palatal major connector. Also, other studies showed that component of framework which is not in close contact with abutment teeth is occlusal rest [9].

Another study showed that the mechanical properties as flexural strength, impact strength, and hardness values of 3D-printed resin by aging have inferior properties and this may affect retention at center and peripheries of defect and accordingly affect accuracy of fit at this region [10].

Till today, most of the previously published articles were concerned of fabricating digital obturators without studying their validity and properties and there is shortage in studies concerning with the effect of aging on tissue adaptability and retention of digital obturator. The aim of this study is to evaluate the effect of aging on tissue adaptation and retention of the digitally fabricated obturator. The null hypothesis is that there is no effect of aging on retention and tissue surface adaptation on digital manufacturing of obturators.

## Methods

### Study setting

This experimental study was conducted in the prosthodontic department and the CAD/CAM laboratory, Faculty of Dentistry Tanta University.

### Ethical consideration

Approval of this research was obtained from the Research Ethics Committee (REC), Faculty of Dentistry, Tanta University. The design and procedures of the present study were accomplished according to the research guidelines adopted by REC, Faculty of Dentistry, Tanta University.

### Sample size of the study

The minimum number of sample size for this study is seven samples. The significance level was $\:0.05$ and the power sample size was more than $\:\:80\text{\%}$ for this study and the confidence interval 95% and the actual power is 99.91%. The sample size was calculated using a computer program G power version 3.

#### 1. Preparation of the cast

A previously fabricated maxillary model (Armany Class I) from epoxy resin (**Ramses co.**,** Alexandria**,** Egypt**) was used, four circles (2.0 × 2.0 mm2 with 2.0 mm height) were created, two points at residual maxilla and the other two points in the defect to serve as landmarks for measurements and analyses by geomagic software. For the design, the clasp was I-bar retentive arm on central incisor & double Aker on first & second molar. Major connector: palatal plate connects parts of framework. Minor connector: meshwork type minor connector was used. Indirect retainer: cingulum rest on canine or occlusal rest on premolar. On the abutment teeth, three occlusal rest seats were prepared.

#### 2. Scanning & Designing the cast

**Fig. 1:**
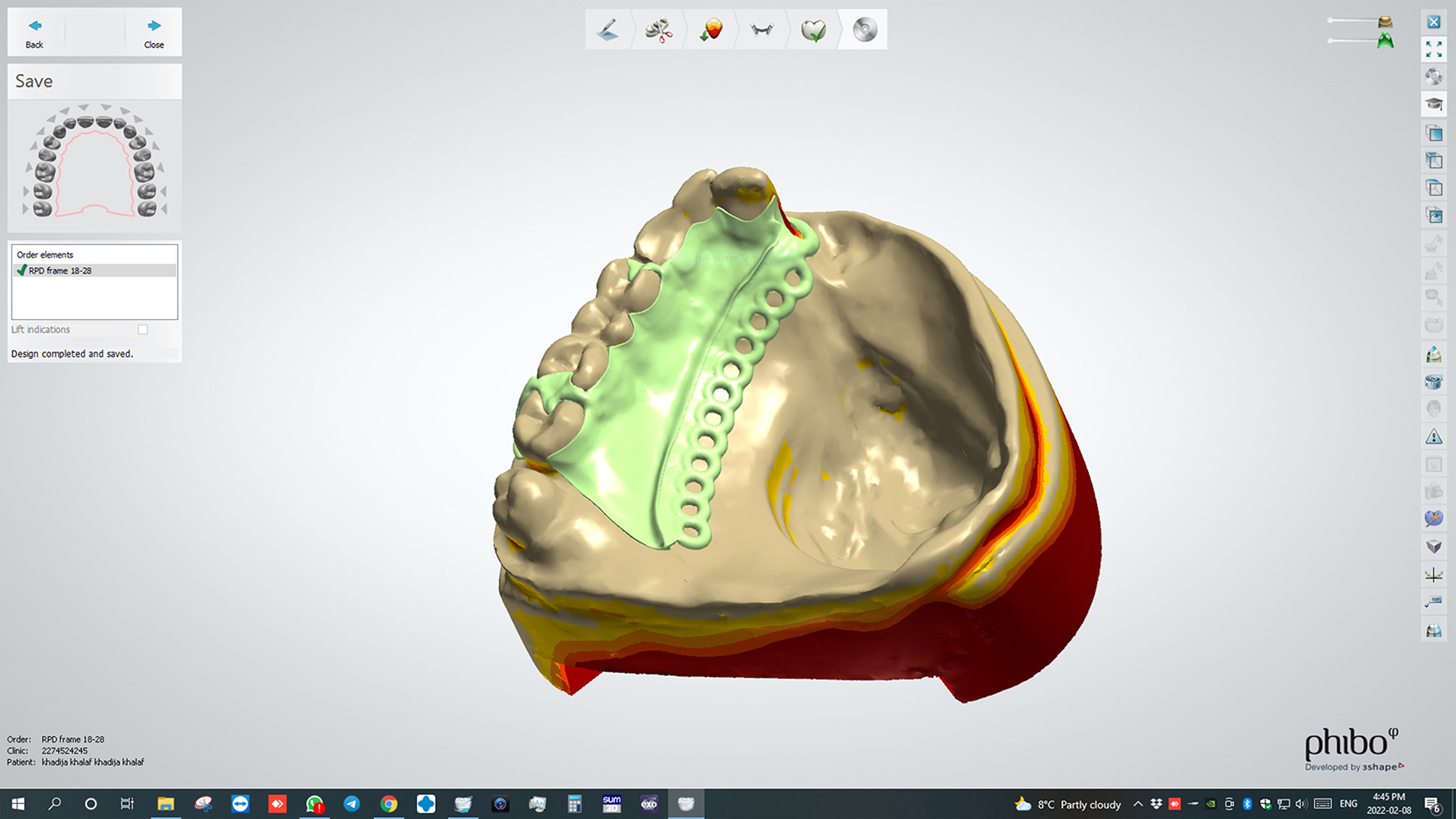
Most frequently reported EQ-5D-5 L profiles

The optical scanner (**DOF – Swing™ Dental Scanner, South Korea**) was used to scan the reference model. The STL file format of the 3D virtual model was then imported to the CAD/CAM software (**3 Shape Dental System; 3 Shape A/S**,** Copenhagen**,** Denmark**) to start digital design. After the 3D framework design was finished, Fig. 1. the file was saved in the form of STL design file then it was sent to the digital light processing-based 3D printer (**Epax Lcd resign 3Dprinter user manual**,** United States of America**), to fabricate resin (**Harz labs Dental Pink 3D Printing Resin**,** Russia)** pattern to check the design and fit of framework before milling. The STL file was transferred to the Dental 5- Axis CNC milling machine (**ED5X Emar dental mills**,5-axis milling machine,** Egypt**) to start the subtractive milling process.

**Fig. 2:**
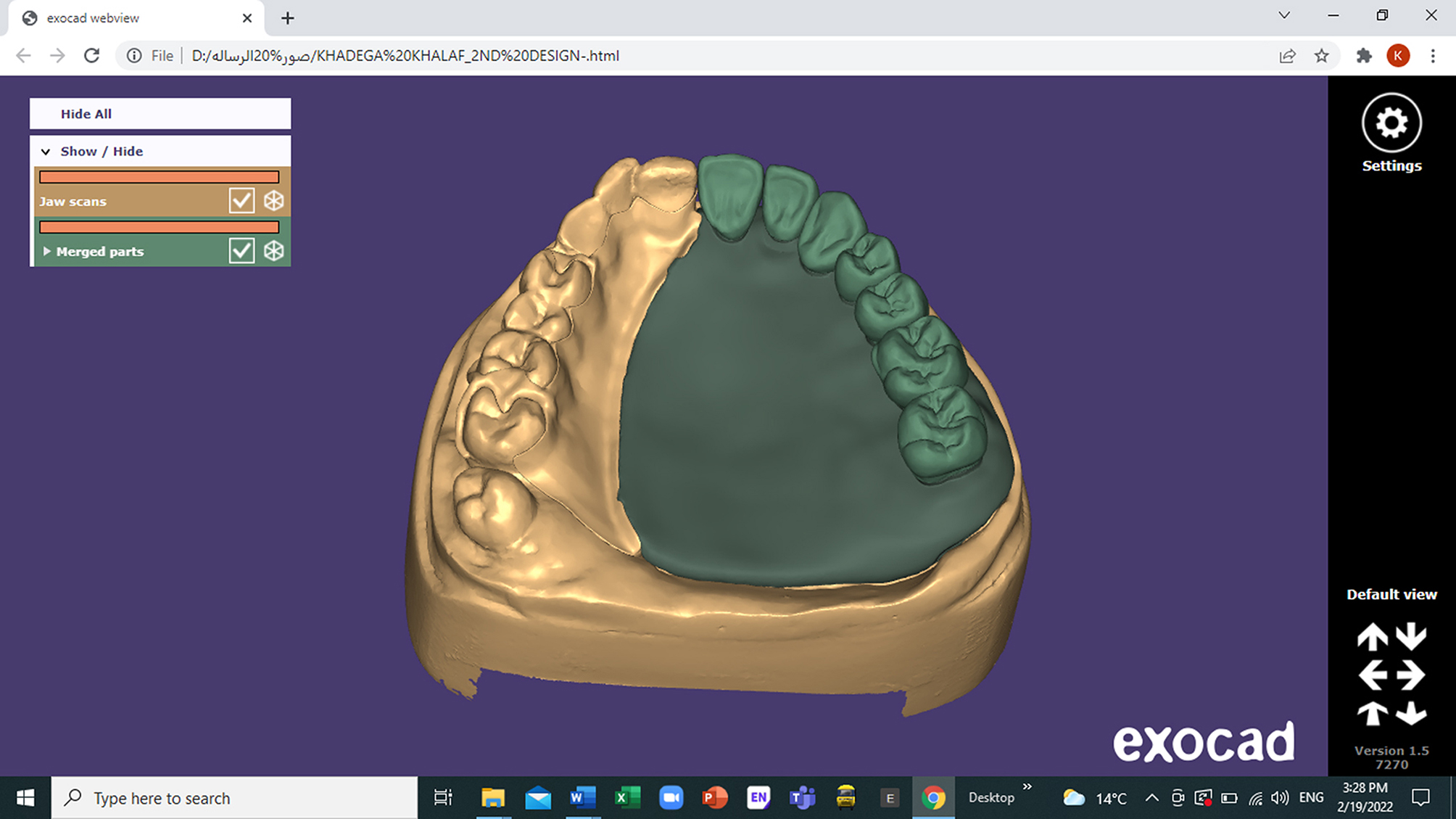
Design of obturator bulb, base and teeth on Exo-CAD software

Reference cast with milled cobalt-chromium (Remanium^®^ Star MD II, Germany) framework was scanned by optical scanner. Design of obturator bulb and obturator base with teeth was made on (**Exocad Dental Cad software**,** Darmstadt**,** Germany**) Fig. 2. The bulb portion was hollowed leaving 2 mm wall thickness all around. The STL file of the obturator bulb was imported to the printer **(Epax Lcd resign 3D printer user manual**,** United States of America**) then 3D printer started printing layer by layer and the light source polymerized it layer by layer. The milled obturator framework was cemented to 3D printed obturator bulb and obturator base with teeth by light cure resin- based cement (Kerr NX3 Light Cured Resin Cement, Italy) and through retention grids.

#### 3. Evaluation of the retention

**Fig. 3:**
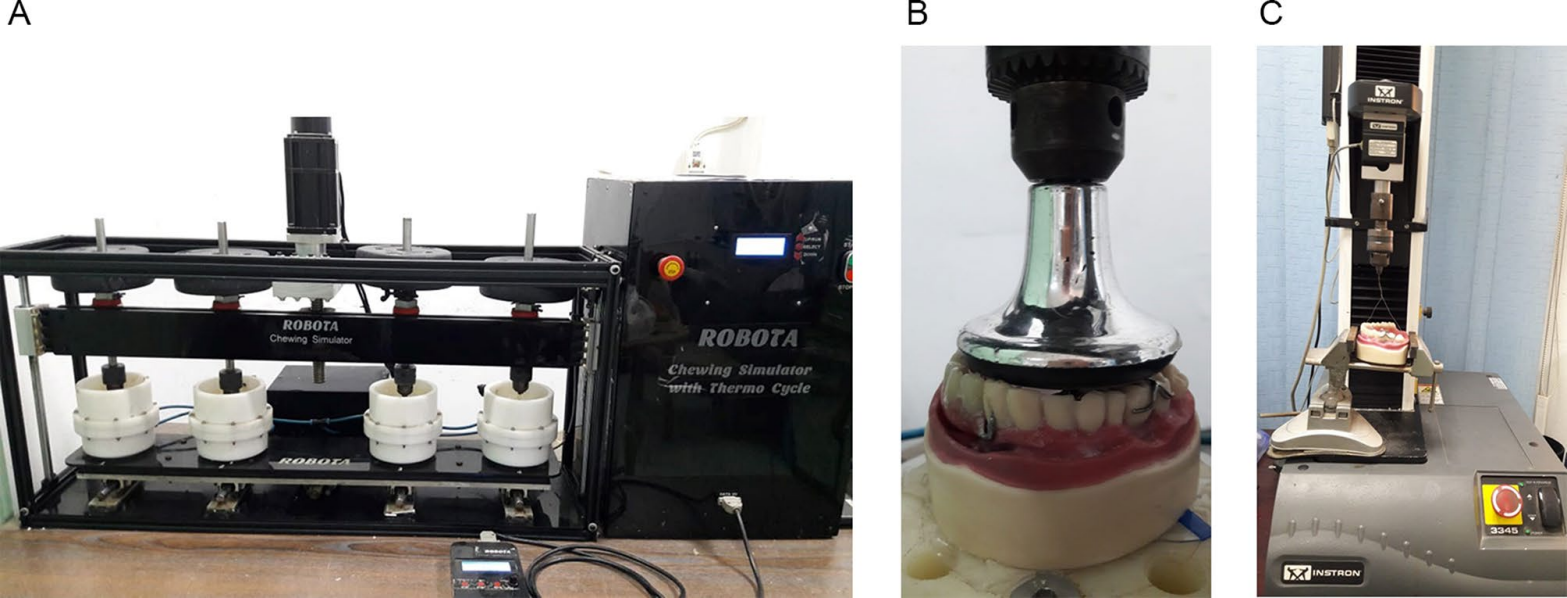
(**a**) Final digital obturator on reference model on ROBOTA chewing simulator (**b**) Circular flat end-shaped plastic load applicator centrally positioned between second premolar and first molars (**c**) Testing machine Instron Instruments allowed the insertion of the clasp to its terminal position and its removal from the abutment

To evaluate the effect of aging on retention of obturator, the cast with the obturator was fixed to the lower fixed compartment of the testing machine (Bluehill^®^ Lite from Instron Instruments). Figure 3a. To do the cyclic loading test, a programmable controlled equipment with four stations multimodal ROBOTA chewing simulator (**Model ACH-09075DC-T**,**AD-TECH TECHNOLOGY CO.**,**LTD.**,**GERMANY**) was used. ROBOTA chewing simulator which has four chambers simulating the vertical and horizontal movements simultaneously in the thermodynamic condition. Each of the chambers consists of an upper Jackob’s chuck as a holder for the vertical screw that can be fixed to the load applicator. Each obturator was then placed on the corresponding model while Jakobe’s chuck of the upper part of machine was fixed through inverted circular flat end-shaped plastic load applicator centrally positioned between second premolar and first molars to facilitate the aligning with the loading axis of machine and proper load distribution. A weight of five kgf., comparable to 49 N of chewing force was applied and the test was repeated 37,500, 75,000 and 150,000 times to simulate clinically 3, 6 and 12 months of chewing condition. Figure 3b.

Testing machine (**Model 3345; Instron Instruments Ltd.**,** USA**) was adjusted to allow the insertion of the clasp to its terminal position and its removal from the abutment which simulating the placement and removal of an obturator that the sample was attached through centrally positioned 0.014’’ mm diameter orthodontic wire to facilitate the aligning with the loading axis of machine and proper load distribution. A tensile load with pull out mode of force by the wire that was attached to upper compartment of materials testing machine at a crosshead speed of 5 mm/min. The load required to totally dislodge sample was recorded in Newton.

Then The test was repeated 360, 720 and 1440 cycles to simulate clinically the 3, 6 and 12 months. Retention was then evaluated after mechanical test (cyclic loading and insertion removal test) by using Bluehill Lite from Instron Instruments [11] Fig. 3C.

#### 4.Evaluation of the tissue surface adaptation

**Fig. 4:**
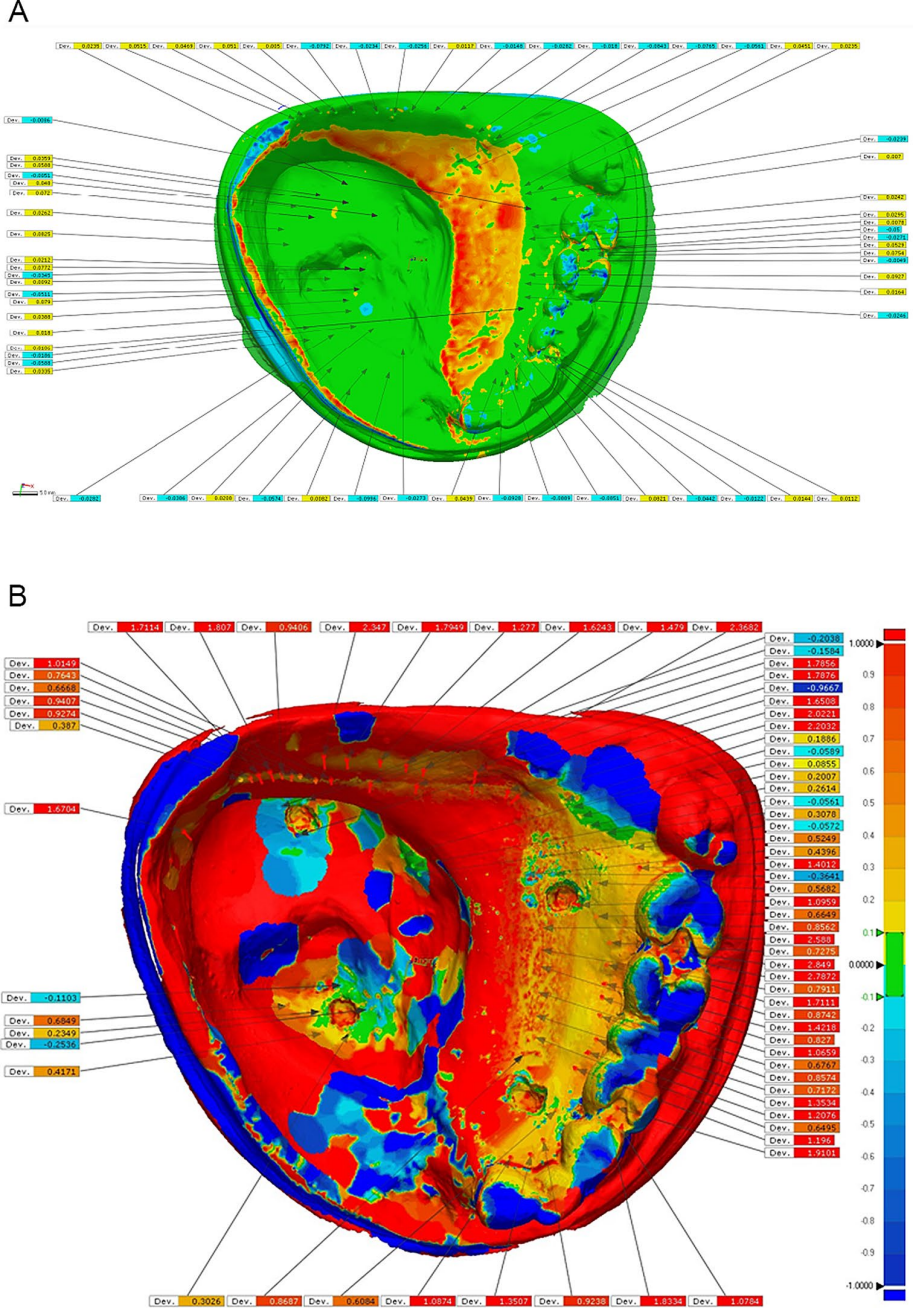
The color surface map of the fitting surface of the measured obturator (**a**) Before application of load (**b**) After application of load

To evaluate tissue surface adaptation, the reference cast and fitting surface of digital obturator were scanned separately using a laboratory scanner (**DOF – Swing Dental Scanner**), The STL file of fitting surface of obturator and the primary model were imported to the surface matching software program **(Geomagic Control X 2018**,** 3D Systems**,** Tokyo**,** Japan**). The STL file of obturator was superimposed onto the STL file of the primary model according to reference points then best fit and 3D compare options were selected as the final matching occurred. The color surface map of the fitting surface of the measured obturator appeared. The selected areas in which the adaptation measurements were performed and represented by a total of sixty points were as follows: The rest/rest seat areas of left first premolar and first and second molar. The major connector area includes the anterior and the posterior strap area. Defect area including central and periphery of defect. The gap distance between the reference cast and the obturator base at the selected areas was measured digitally. Adaptation was then evaluated after mechanical test (cyclic loading and insertion removal test) as the previous method. Figure 4a and b.

### **Data analysis**

Retention and tissue surface adaptation of digital obturator results were examined using Shapiro-Wilk test. The information was parametric and normally distributed.

Data were analyzed using the Statistical Package for the Social Sciences (IBM SPSS Statistics 26), with the Student’s t-test and multi variable ANOVA employed to determine significance.“. P-value < 0.05(*) was considered significant difference.

## Results

**Graph 1:**
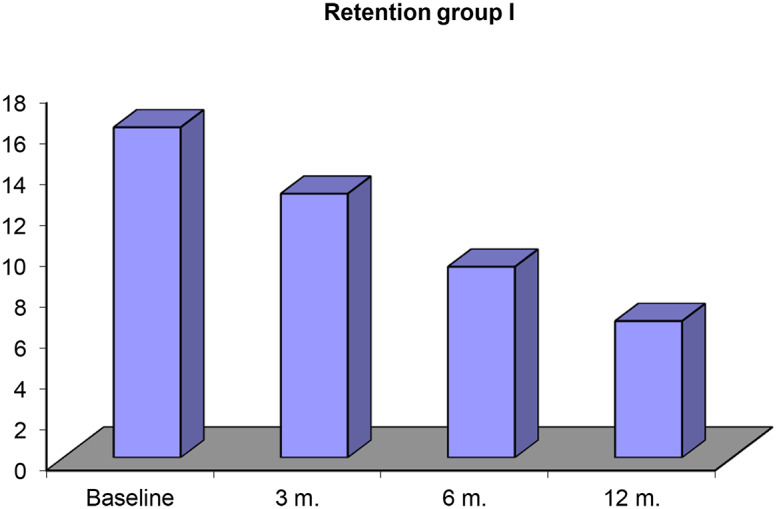
Retention force measured initially and after 3, 6 and12 months for digitally fabricated obturators

In Table 1 and graph 1, mean and standard deviation for retention values and percent of retention reduction, initially as a baseline was (16.18 ± 1.13), after 3 months was (12.93 ± 1.38), after 6 months was (9.35 ± 2.11) and after 12 months was (6.69 ± 0.97) and percent of reduction of retention between initially (baseline) and after 3 months (20.1%), between base line and after 6 months (42.2%) and between base line and after 12 months (58.7%). P-value < 0.05 so significant difference was observed between baseline and 3, 6 and12 months.

**Table 1:** Retention force measured initially and after 3, 6 and12 months for digitally fabricated obturators

Retention		Baseline		3 months.	6 months.		12 months.		
**Range**		14.5–17.8		11.2–15.1	6.05–12.1		5.2–8.2		
**Mean ± SD**		16.18 ± 1.13		12.93 ± 1.38	9.35 ± 2.11		6.69 ± 0.97		
**% Of reduction**				20.1%	42.2%		58.7%		
**F test**							56.048		
**P value**					0.001*				
**Baseline & 3 m.**	**Baseline & 6 m.**		**Baseline & 12 m.**			**3 m. & 6 m.**		**3 m. & 12 m.**	**6 m. & 12 m.**
0.001*	0.001*		0.001*			0.001*		0.001*	0.002*

p-value 0.001* between **Baseline & 3 m**

p-value 0.001* between **Baseline & 6 m**

p-value 0.001* between **Baseline & 6 m**

p-value 0.001* between **3 m & 6 m**

p-value 0.001* between **3 m & 12 m**

p-value 0.002* between **6 m & 12 m**

P-value < 0.05(*) was considered significant difference

In Table 2 and graph 1 for tissue surface adaptation test, At rest area, the Mean ± SD of deviation gap distance before application of load is (0.0074 ± 0.0015) and after application of load is (2.0494 ± 0.1734). At major connector area, The Mean ± S.D of deviation gap distance before application of load is (0.0178 ± 0.0059) and after application of load is (0.5699 ± 0.1021).At center and periphery of defect, The Mean ± S.D of deviation gap distance before application of load is (0.0329 ± 0.0395) and after application of load is (0.4103 ± 0.0844). For the overall gap distance, the Mean ± S.D of gap distance before application of load (0.15 ± 0.1943) and after application of load (0.9874 ± 0.1015). P-value < 0.05 showed that there was a significant difference at all measured areas before and after application of load.

**Table 2:** The overall fit of the obturator to the reference model before and after application of load

		Before	After	t. test	*p*. value
**Rest**	**Range**	0.0045–0.0088	1.874–2.287	31.162	0.001*
	**Mean ± SD**	0.0074 ± 0.0015	2.0494 ± 0.1734		
**Major connector**	**Range**	0.0099–0.0242	0.4056–0.7152	14.282	0.001*
	**Mean ± SD**	0.0178 ± 0.0059	0.5699 ± 0.1021		
**Center and peripheries of defect**	**Range**	0.0105–0.1215	1.468–1.835	32.523	0.001*
	**Mean ± SD**	0.0329 ± 0.0395	1.6693 ± 0.1271		
**The overall mean accuracy of fit**	**Range**	0.061–0.59	0.8541–1.1354	10.107	0.001*
	**Mean ± SD**	0.15 ± 0.1943	0.9874 ± 0.1015		

P_value 0.001* for The overall fit before and after application of load at rest

P_value 0.001* for The overall fit before and after application of load at **Major connector**

P_value 0.001* for The overall fit before and after application of load **Center and peripheries of defect**

P-value < 0.05(*) was considered significant difference

**Graph 2:**
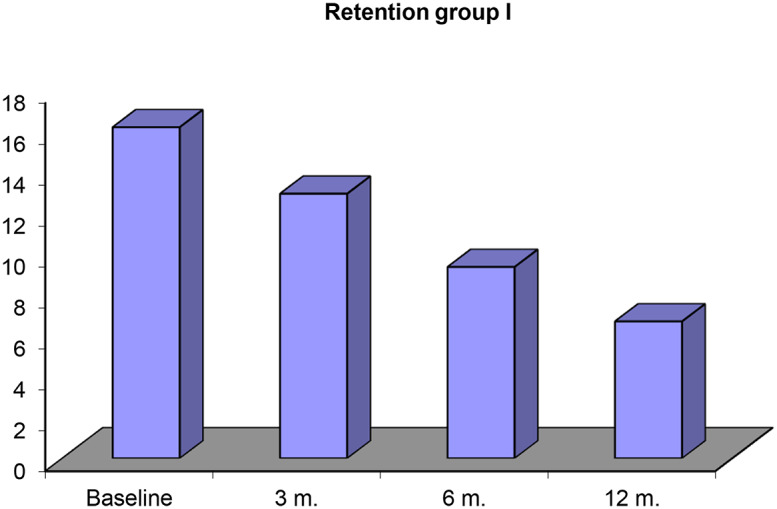
The overall fit of the obturator to the reference model before and after application of load

## Discussion

Because of the disadvantages of traditional method, the full digitalization is used which leads to favorable clinical outcomes, better retention, fewer patient visits, better material properties and biocompatibility. identification of anatomical landmarks on digitized casts, easy data storage and production of duplicated obturators, reduce hazards of infectious cross contamination, It allows application of provisions for esthetic designs, excellent precision of fit and longevity [4].

A researcher used CAD/CAM for construction process and created a digitized database of fabricated acrylic obturators to be kept for patients’ emergency needs. Their results presented that the obturator can be successfully scanned in spite of its structural complexity, modeled as three-dimensional data and stored in the digital system [12].

Tasopoulos et al, [3] described construction of two-piece definitive obturator for completely edentulous patient using 3D printing of large palatal defect using a CT scan data. The printed model was used for making a wax pattern for the extensive nasal part without making an impression to the defect region. The pattern was then placed in the patient’s mouth on which the following steps were completed in conventional manner.

In this study, the principal model with (Armany class I classification) was chosen as it was declared that these defects always considered to be challenging cases due to the problem of stability and retention of the obturators [1].

The framework of CAD-CAM obturator was fully manufactured by the direct technique (milling and 3D printing). Direct CAD-CAM technique proved to be better than those fabricated by the indirect technique in overall fit values [13].

The obturator framework was fabricated by subtractive milling technique from Cobalt-chromium alloy as it is considered the standard materials for CAD-CAM frameworks and to overcome casting inaccuracies, limitations, [14]and improve the overall framework fit as well as the fit of occlusal rests [15]. The 3D printing manufacturing was used for bulb fabrication in this study because CAD-CAM PMMA showed superior characteristics in its flexural strength, hardness, impact strength and flexural modulus [16]. Recently, a study declared that mechanical properties have relation with dimensional accuracy that 3D-printed resin had inferior flexural strength, impact strength, and hardness values than heat-polymerized resin after application of load and this may affect retention at center and peripheries of defect and accordingly affect accuracy of fit at this region [10].

The hollow obturator base was 3D printed in this study due to excellent accuracy of 3D printers. As well as additive manufacturing has the advantage of producing large objects like facial prostheses with surface irregularities, undercuts, voids and hollow morphology [17].

The chewing cycles and insertion-removal cycles were performed using the chewing simulator due to its ability to reproduce the complex oral environment even to a limited extent, provide information about different materials and ability to maintain their properties during function [18]. Three hundred and sixty (360) repeated insertion and removal cycles were performed to simulate 3 months of clinical function of the obturator. This number was estimated to simulate three daily removals and insertions of the obturator for purpose of hygiene under normal conditions in the morning, after lunch and after dinner and total of 75,000 chewing cycles were applied to clinically simulate six –months. A weight of 5 kgf (49 N) was applied onto the specimens, which is equal to average chewing force [19].

In this present study, software superimposition of framework fabrication and design data was used because it enabled quantitative, comprehensive evaluation of accuracy. Presenting the data as color maps also enabled 3D evaluation of the direction of displacement [9].

In this study, Initial retention as a baseline showed that digital obturator had good retention. This result was the same with a study reported significantly higher retention for digital complete dentures than for conventional ones and this may be due to the weight of the obturator has a significant role in retention and stability which was 28 mg [20]. This matching that, the overall misfit or gap distance in digital obturator is low. This means that the tissue surface adaptation is accepted in the digitally fabricated obturator so this could affect retention of the obturator prosthesis. The result of this study agrees with the result of Neena et al., which showed that CAD/CAM obturators showed lower overall misfit values. On the other hand, the result of this study differs from another study found that CAD frameworks exhibited the highest discrepancies due to inaccuracies during scanning of arches using digital scanners or induced software errors while processing the STL files [21].

The observed decrease in retention in this study may be due to two factors. The first one is that the withdrawal of the obturator from the defect area reduces retention of buccally placed clasps, the selected case has a larger area of the defect so less tissue to support the prosthesis and fewer teeth, so; vertical extension was large enough to cover the entire defect and led to decreased retention. The second cause for retention loss is the design of digital obturator in this study; the major connector ended around defect due to milling block thickness (< 25 mm). Thus, the prosthesis could not be milled in proper dimensions, so modification of the palatal contour of the obturator was made and became shallower to be compatible according to the blank height so ended up with decreased retention [22].

After application of insertion removal cycles and chewing cycles, the overall fit and tissue surface adaptation were decreased. This may be a multifactorial decrease due to surface adaptation of 3D printed obturator bulb, build angle, thickness of printing layers, types of 3D printer used and/or type of resin used. All these factors may affect the accuracy of a 3D printed denture or obturator prosthesis bases [23].

The digital obturator can be used successfully with the shortcomings of loosening retention and adaptation So, clinical trials should investigate the clinical acceptance of these shortcomings and limitation of digital scanning of severe under cut and deep palatal defect.

Authers tried to generate 3D digital casts of maxillary defects including the maxillary dentition and the defective region based on multisource data registration and evaluated their clinical efficiency. The maxillofacial region was scanned using CT and the maxillary arch and palate were scanned using an intraoral optical scanner. The 3D images from the CT and intraoral scanner were registered and merged to form a 3D digital cast of the maxillary defect containing the anatomic structures. They concluded that these digital casts were convenient with conventional stone casts in accuracy and were suitable for clinical use [3].

### Limitations

1. Setting of teeth was done according to mirror image with the other side due to there is no mandibular cast as an opposing model.
2. limitation of digital scanning of severe under cut and deep palatal defect.

## Conclusions

1. Digitally designed and fabricated obturator obtains the characteristics of high retention and high tissue surface adaptation after fabrication and before application of load.
2. Retention and tissue surface adaptability decreases upon application of load and aging process.

## Data Availability

On reasonable request, the datasets utilized or analyzed during the present study are accessible from corresponding author.

## Abbreviations

CAD: Computer-aided design
CAM: Computer-aided manufacturing
3D: Three-dimensional
STL: Standard tessellation language
SM: Subtractive milling
AM: Additive manufacturing
CNC: Computer numeric control
SPSS: Statistical Package for Social Sciences
